# Establishment and validation of a clinical nomogram model based on serum YKL-40 to predict major adverse cardiovascular events during hospitalization in patients with acute ST-segment elevation myocardial infarction

**DOI:** 10.3389/fmed.2023.1158005

**Published:** 2023-05-22

**Authors:** Caoyang Fang, Jun Li, Wei Wang, Yuqi Wang, Zhenfei Chen, Jing Zhang

**Affiliations:** ^1^Department of Cardiology, The Second People's Hospital of Hefei, Hefei Hospital Affiliated to Anhui Medical University, Hefei, Anhui, China; ^2^Graduate School, Bengbu Medical College, Bengbu, Anhui, China; ^3^Department of Cardiology, The Lu’an Hospital Affiliated to Anhui Medical University, Lu’an, Anhui, China; ^4^Department of Cardiology, The Lu’an People's Hospital, Lu’an, Anhui, China

**Keywords:** serum YKL-40, acute ST-elevation myocardial infarction, STEMI, adverse cardiovascular events, MACE, nomogram

## Abstract

**Objective:**

This study aimed to investigate the predictive value of a clinical nomogram model based on serum YKL-40 for major adverse cardiovascular events (MACE) during hospitalization in patients with acute ST-segment elevation myocardial infarction (STEMI).

**Methods:**

In this study, 295 STEMI patients from October 2020 to March 2023 in the Second People’s Hospital of Hefei were randomly divided into a training group (*n* = 206) and a validation group (*n* = 89). Machine learning random forest model was used to select important variables and multivariate logistic regression was included to analyze the influencing factors of in-hospital MACE in STEMI patients; a nomogram model was constructed and the discrimination, calibration, and clinical effectiveness of the model were verified.

**Results:**

According to the results of random forest and multivariate analysis, we identified serum YKL-40, albumin, blood glucose, hemoglobin, LVEF, and uric acid as independent predictors of in-hospital MACE in STEMI patients. Using the above parameters to establish a nomogram, the model C-index was 0.843 (95% CI: 0.79–0.897) in the training group; the model C-index was 0.863 (95% CI: 0.789–0.936) in the validation group, with good predictive power; the AUC (0.843) in the training group was greater than the TIMI risk score (0.648), *p* < 0.05; and the AUC (0.863) in the validation group was greater than the TIMI risk score (0.795). The calibration curve showed good predictive values and observed values of the nomogram; the DCA results showed that the graph had a high clinical application value.

**Conclusion:**

In conclusion, we constructed and validated a nomogram based on serum YKL-40 to predict the risk of in-hospital MACE in STEMI patients. This model can provide a scientific reference for predicting the occurrence of in-hospital MACE and improving the prognosis of STEMI patients.

## 1. Introduction

Acute ST-segment elevation myocardial infarction (STEMI) is an acute severe heart disease caused by the rupture of vulnerable atherosclerotic plaques in coronary vessels. At present, the key to the treatment of STEMI lies in dredging the blocked vessels and restoring the blood supply to the myocardium. Existing treatments are mainly percutaneous coronary intervention (PCI), however, myocardial reperfusion injury often occurs in STEMI patients after PCI with the recovery of myocardial blood supply, leading to the occurrence of major cardiovascular adverse events (MACEs) such as heart failure, malignant arrhythmia, and even sudden death, with a poor prognosis (1, 2). In addition to thrombosis and platelet activation, several studies have shown that the inflammatory response plays an important role in the development and progression of STEMI (3). Therefore, how to predict the occurrence of MACE after PCI in STEMI patients early is of great clinical significance for improving the prognosis.

Serum YKL-40 is a glycoprotein belonging to the chitinase-similar protein family, also known as human glycochondroprotein 39 (HC-gp39) and chitinase 3-like protein 1 (4). YKL-40 can be secreted by a variety of cells, including monocytes, macrophages, neutrophils, chondrocytes, and vascular smooth muscle cells (5–7). To date, serum YKL-40 has been implicated as a novel inflammatory marker contributing to cell proliferation and differentiation, angiogenesis, and tissue remodeling (8). It has been shown that serum YKL-40 is closely associated with the early and late stages of the atherosclerotic process, and YKL-40 induces monocytes to mature into macrophages, which are then secreted by macrophages and activated macrophages at a later stage of differentiation (9).In addition, previous studies have shown that YKL-40 levels are higher in patients with myocardial infarction (10), stable coronary artery disease (11, 12), and heart failure (13). High levels of serum YKL-40 are an independent predictor of MACE after PCI in STEMI patients, and serum YKL-40 can be used as a biomarker to predict the long-term prognosis of STEMI patients after PCI (14, 15). Serum YKL-40 levels predict postoperative myocardial reperfusion and in-hospital MACE in STEMI patients (16).

Currently, the most effective treatment for STEMI patients remains percutaneous coronary intervention. However, the incidence of major adverse cardiac events (MACEs) during hospitalization after PCI varies according to different risk factors for STEMI, particularly in high-risk patients (17, 18). Predictive models will allow physicians to better identify high-risk patients, which will facilitate a more personalized approach to managing these cases.

As a simple and accurate visualization tool, nomograms have been widely used to predict the incidence of each patient endpoint event. Developed a simple and practical nomogram to predict the prognosis of ACS patients with potential clinical value (19). However, few studies predict serum YKL-40 with nomograms and in-hospital MACE in STEMI patients. Therefore, our study aimed to develop and validate a nomogram, combined with clinical characteristics to predict the likelihood of in-hospital MACE in STEMI patients with serum YKL-40, to provide a scientific reference for clinical treatment decisions and the prevention of in-hospital MACE in STEMI patients.

## 2. Methods

### 2.1. Study population

We enrolled 295 patients with STEMI who underwent PCI between October 2020 and March 2023 in the Second People’s Hospital of Hefei. Inclusion criteria were STEMI diagnosis (17): (1) chest pain symptoms within 24 h before admission, lasting >30 min; (2) ST-segment elevation and/or abnormal Q waves in ≥2 consecutive leads suggested by electrocardiogram; (3) serum biochemical markers creatine kinase isoenzyme (CK-MB) and/or cardiac troponin T (cTnT) increased within 24 h after the onset of chest pain. Exclusion criteria: (1) malignant tumor; (2) blood system diseases; (3) active infection; (4) chronic inflammatory diseases; (5) severe liver or kidney dysfunction; (6) chronic heart failure; (7) use of steroids or chemotherapeutic drugs. This study was approved by the Ethics Committee of the Second People’s Hospital of Hefei, and all patients signed an informed consent form.

### 2.2. Detection of serum YKL-40

Five milliliters of cubital venous blood were taken from all STEMI patients before emergency PCI in a common biochemical tube, centrifuged at a centrifugal radius of 15 cm, 2,500 r/min, for 10 min at 4°C, and the separated serum was placed in a sterile EP tube and stored in a −80°C freezer until testing. Specimens were tested in the same batch and thawed thoroughly and uniformly at room temperature for the first time. Serum YKL-40 levels were measured by enzyme-linked immunosorbent assay (ELISA), and a standard curve was drawn to determine the YKL-40 concentration of unknown samples, with intra-batch and inter-batch coefficients of variation of <6 and 8%, respectively. The operation process shall be carried out in strict accordance with the requirements in the instructions for use.

### 2.3. General data and laboratory tests

General data collection: The general conditions and vital signs of the patients at admission were recorded by consulting the medical records through the electronic case system of the hospital, including age, gender, BMI, diabetes, hypertension, smoking, family history of coronary heart disease, heart rate, systolic blood pressure, diastolic blood pressure, Killip class and Gensini score; the laboratory parameters of fasting tests on the second day after admission included neutrophils, hemoglobin, platelets, total cholesterol, triglycerides, LDL-C, HDL-C, fasting blood glucose, serum creatinine, blood uric acid, and albumin in all patients.

### 2.4. Definition and grouping of MACE events

MACE defined the primary endpoint event as all-cause mortality. Secondary endpoints were reinfarction, emergency revascularization, cardiac arrest, acute heart failure, cardiogenic shock, malignant arrhythmia (including ventricular tachycardia/ventricular fibrillation, sinus arrest, high-grade or third-degree atrioventricular block), mechanical complications of myocardial infarction, stroke, and severe bleeding (hemoglobin decrease ≥3 g/L). Among them, 113 STEMI patients developed MACE and 182 STEMI patients did not develop MACE events. STEMI patients were randomly divided into a training group (*n* = 206) and a validation group (*n* = 89) according to 7:3.

### 2.5. Random forest model variables selection

Random forest (RF) is a learning method consisting of many individual decision trees that operate as a collection. In the classification task, each tree in RF produces a class prediction, and the class (mode) that votes the most becomes the overall model prediction (18). Each decision tree node uses a subset of features randomly selected from the entire feature list. Increasing the number of trees in the forest to find the number of trees without more significant performance gain. The number of random features to consider in each tree is called MTRYS. Lower Mtry values lead to more differences, fewer associated trees, and yield better stability. In addition, low MTRY also tends to make better use of variables that have moderate effects on response variables. However, lower MTRY values also lead to the worse mean performance of trees as they are constructed based on suboptimal variables. Finally, the minimum size of a terminal node (the number of patients classified at that node) is called the node size. Setting this number more often leads to smaller tree growths. The hyperparameters of all models (MTRYS, minimum number of trees, total number of trees) were adjusted using a grid search method.

### 2.6. Development and validation of a nomogram

A nomogram was developed based on variables selected by the random forest model and multivariate logistic regression analysis. Receiver operating characteristic (ROC) area under the curve (AUC) and model C-index were used to assess the performance of the model; a calibration curve was drawn to evaluate the calibration of the model; a decision curve was drawn to analyze the net benefit rate of this nomogram model in predicting in-hospital MACE in STEMI patients. ROC curves were plotted to assess the efficacy of nomogram models and TIMI scores in predicting in-hospital MACE in STEMI patients.

### 2.7. Statistical methods

Statistics and graphs were performed using SPSS 26.0 and GraphPad Prism9.0. Kolmogorov–Smirnov normality test was performed for measurement data, which conformed to the normal distribution and was expressed as mean ± standard deviation and independent sample t-test was used for comparison between the two groups; measurement data without normal distribution were expressed as median M (P25, P75), and Mann–Whitney U test was used for comparison between the two groups; enumeration data adoption rate was expressed, and chi-square test was used for comparison between the two groups; RF model variable selection and nomogram development and validation were statistically analyzed based on R 4.2.2. All statistics were performed using two-sided tests, and *p* < 0.05 was considered statistically significant.

## 3. Results

### 3.1. Comparison of clinical data between the training set and validation set

A total of 295 STEMI patients treated with PCI were included in this study. They were randomly divided into the training group (*n* = 206) and the validation group (*n* = 89), 115 males (55.8%) were in the training group, with a mean age of 63.22 ± 13.39; 62 males (69.7%) in the validation group, with a mean age of 62.27 ± 15.01. There was no statistical difference between the training group and the validation group except for gender (*p* < 0.05), as shown in Table 1.

**Table 1:** Comparison of general clinical data between Training set and Validation Set [Mean ± standard deviation, M (P_25_,P_75_), number of cases and percentage (*n*%)].

Variables	Total (*n* = 295)	Training set (*n* = 206)	Validation set (*n* = 89)	*p*-value
Age, years	62.93 ± 13.88	63.22 ± 13.39	62.27 ± 15.01	0.591
Male, *n* (%)	177 (0.6)	115 (55.8)	62 (69.7)	0.026^*^
BMI, kg/m^2^	24.27 ± 3.56	24.18 ± 3.48	24.27 ± 3.74	0.525
Hypertension, *n* (%)	146 (49.5)	100 (48.5)	46 (51.7)	0.62
Diabetes, *n* (%)	85 (28.8)	56 (27.2)	29 (32.2)	0.347
Smoking, *n* (%)	152 (51.5)	101 (49)	51 (57.3)	0.192
Family histories of CHD, *n* (%)	39 (13.2)	27 (13.1)	12 (13.5)	0.93
Systolic BP, mmHg	122.75 ± 26.65	122.64 ± 27.11	123.01 ± 25.7	0.912
Diastolic BP, mmHg	70 (61, 83)	70 (60, 80.25)	71 (64, 86.5)	0.073^a^
Killip>2, *n* (%)	90 (30.5)	59 (28.6)	31 (34.8)	0.289
TG, mmol/L	1.51 (1.05, 2.1)	1.51 (1.02, 2.08)	1.61 (1.07, 2.37)	0.265^a^
TC, mmol/L	4.51 ± 1.03	4.44 ± 0.96	4.68 ± 1.17	0.068
HDL-C, mmol/L	1.06 (0.91, 1.22)	1.07 (0.92, 1.22)	1.04 (0.89, 1.24)	0.97^a^
LDL-C, mmol/L	2.93 ± 0.85	2.88 ± 0.8	3.05 ± 0.96	0.116
Creatinine, umol/L	72.6 (60, 88.8)	73.25 (60.23, 87.6)	72.3 (58.4, 90.4)	0.918^a^
Uric acid, umol/L	362.2 (293.6, 433.1)	360.95 (286, 431.23)	373.6 (304.9, 436.75)	0.389^a^
Glucose, mmol/L	7.21 (5.71, 9.53)	7.2 (5.66, 9.53)	7.34 (5.8, 9.66)	0.575^a^
Neutrophils, ×10^9^/L	7.91 (5.95, 10.31)	8.25 (5.89, 10.66)	7.53 (5.96, 9.7)	0.114^a^
Hemoglobin, g/L	135 ± 19.46	135.31 ± 19.53	134.3 ± 19.38	0.682
Platelets, ×10^9^/L	205.1 (164, 252.1)	211.5 (157.5, 253)	200 (167.5, 250)	0.897^a^
Albumin, g/L	38.1 (35.4, 40.3)	38.4 (35.4, 40.43)	37.8 (35, 39.4)	0.371^a^
LVEF	58 (52, 62)	58 (52, 63)	58 (52, 62)	0.837^a^
Gensini score	64 (42, 90)	66 (40, 90)	58 (44, 88)	0.592^a^
Serum YKL-40, ng/dL	1227.58 (729.53, 1561.52)	1243.76 (822.01, 1562.64)	1206.32 (678.09, 1601.53)	0.51^a^

BMI, body mass index; CHD, coronary heart disease; BP, blood pressure; TG, triglyceride; TC, total cholesterol; LDL-C, low-density lipoprotein cholesterol C; HDL-C, high-density lipoprotein cholesterol C; LVEF, left ventricular ejection fraction; ^a^Non-normally distributed data are expressed as median M (P25, P75); ^*^p < 0.05; 1 mmHg = 0.133 kPa.

### 3.2. Comparison of STEMI patients with and without MACE events

Compared with the non-MACE group, the serum YKL-40 level was higher in the MACE group (*p* < 0.05; Table 1); there was a significant difference in age between the two groups; there was a significant difference in systolic blood pressure, diastolic blood pressure, TC, HDL-C, creatinine, uric acid, neutrophils, blood glucose, hemoglobin, and LVEF (*p* < 0.05); there was no significant difference in gender, BMI, hypertension, diabetes, current smoking, Killip class of family history of coronary heart disease, TG, LDL-C, platelets, albumin, and Gensini score related indicators between the two groups (*p* > 0.05), as shown in Table 2.

**Table 2:** Comparison of general clinical data between MACE group and non-MACE group [mean ± standard deviation, M (P_25_, P_75_), number of cases and percentage (*n*%)].

Variables	Total (*n* = 295)	MACE group (*n* = 113)	Non-MACE group (*n* = 182)	*p*-value
Age, years	62.93 ± 13.88	66.04 ± 13.02	61 ± 14.08	0.002^*^
Male, *n* (%)	177 (0.6)	71 (62.8)	106 (58.2)	0.434
BMI, kg/m^2^	24.27 ± 3.56	24.01 ± 3.71	24.43 ± 3.46	0.325
Hypertension, *n* (%)	146 (49.5)	54 (47.8)	92 (50.5)	0.645
Diabetes, *n* (%)	85 (28.8)	36 (31.9)	49 (26.9)	0.363
Smoking, *n* (%)	152 (51.5)	60 (53.1)	92 (50.5)	0.67
Family histories of CHD, *n* (%)	39 (13.2)	20 (17.7)	19 (10.4)	0.074
Systolic BP, mmHg	122.75 ± 26.65	118.14 ± 26.08	125.61 ± 26.67	0.019^*^
Diastolic BP, mmHg	70 (61, 83)	70 (60, 80)	71 (62.75, 86)	0.042^a*^
Killip>2, *n* (%)	90 (30.5)	38 (33.6)	52 (28.6)	0.359
TG, mmol/L	1.51 (1.05, 2.1)	1.49 (1.07, 2.23)	1.52 (1.04, 2.09)	0.82^a^
TC, mmol/L	4.51 ± 1.03	4.35 ± 1.02	4.61 ± 1.03	0.034^*^
HDL-C, mmol/L	1.06 (0.91, 1.22)	1.11 (0.93, 1.31)	1.04 (0.91, 1.17)	0.02^a*^
LDL-C, mmol/L	2.93 ± 0.85	2.78 (2.14, 3.44)	2.95 (2.37, 3.55)	0.175
Creatinine, umol/L	72.6 (60, 88.8)	76 (59.9, 100.15)	70.8 (59.8, 82.03)	0.019^a*^
Uric acid, umol/L	362.2 (293.6, 433.1)	382 (325.3, 438.55)	351.3 (277.78, 426.28)	0.033^a*^
Glucose, mmol/L	7.21 (5.71, 9.53)	8.02 (6.46, 10.45)	6.61 (5.36, 8.33)	<0.001^a*^
Neutrophils, ×10^9^/L	7.91 (5.95, 10.31)	8.57 (6.37, 10.89)	7.6 (5.47, 10.02)	0.044^a*^
Hemoglobin, g/L	135 ± 19.46	128.27 ± 17.22	139.19 ± 19.64	<0.001^*^
Platelets, ×10^9^/L	205.1 (164, 252.1)	198 (159.4, 243)	211.3 (167.5, 253.85)	0.223^a^
Albumin, g/L	38.1 (35.4, 40.3)	38.2 (34.1, 39.9)	38 (35.58, 40.43)	0.142^a^
LVEF	58 (52, 62)	55 (48.5, 61)	60 (53.75, 63)	<0.001^a*^
Gensini Score	64 (42, 90)	66 (40, 99.25)	64 (43.5, 84.25)	0.244^a^
Serum YKL-40,ng/dL	1227.58 (729.53, 1561.52)	1501.26 (1245.51, 1753.34)	930.79 (543.31, 1405.38)	<0.001^a*^

BMI, body mass index; CHD, coronary heart disease; BP, blood pressure; TG, triglyceride; TC, total cholesterol; LDL-C, low-density lipoprotein cholesterol C; HDL-C, high-density lipoprotein cholesterol C; LVEF, left ventricular ejection fraction; ^a^Non-normally distributed data are expressed as median M (P25, P75); ^*^p < 0.05; 1 mmHg = 0.133 kPa.

### 3.3. Development and validation of nomograms based on serum YKL-40 for predicting in-hospital MACE in STEMI patients

#### 3.3.1. Selection of RF model variables and multivariate logistic regression analysis

**Figure 1:**
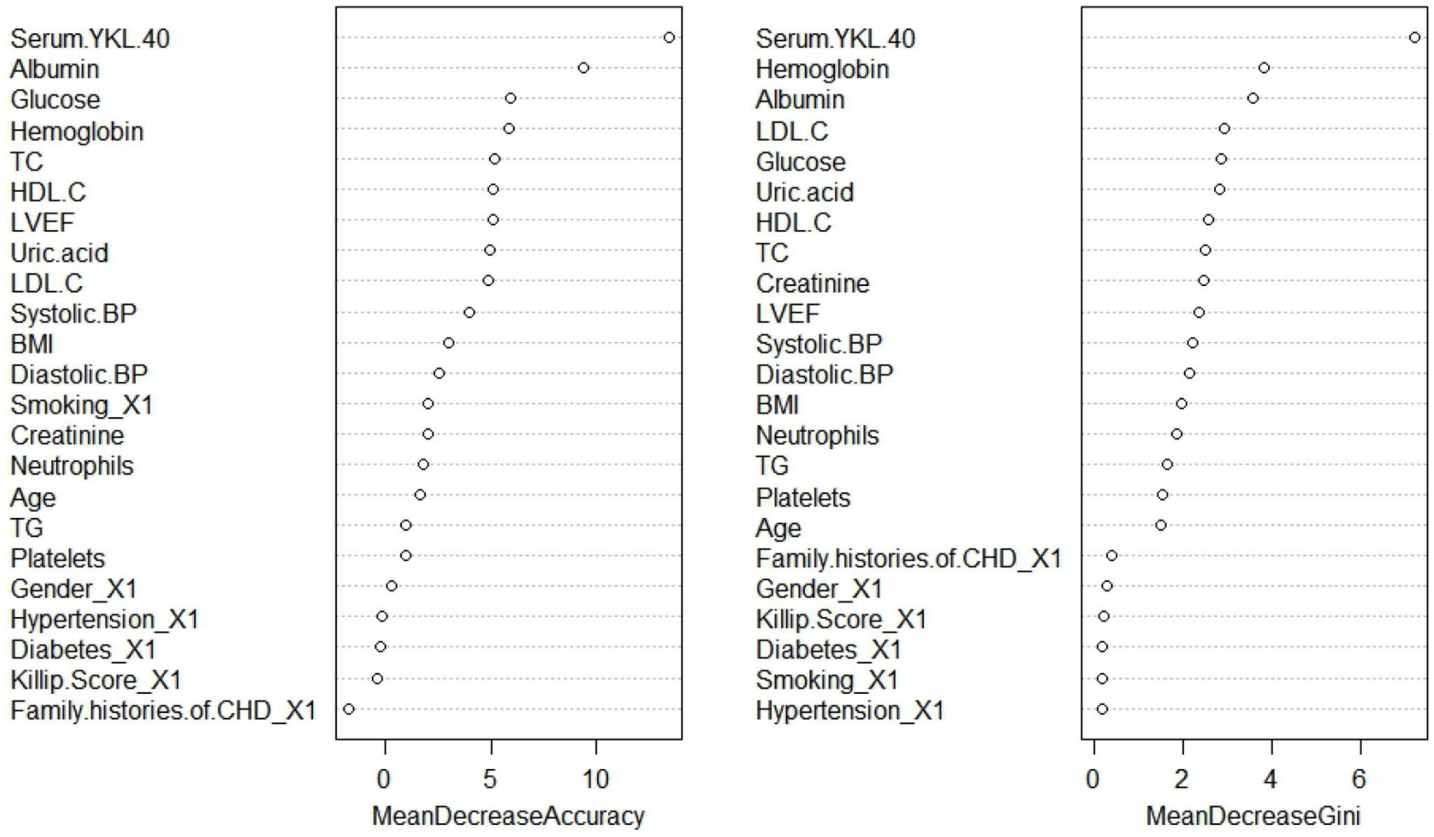
Random forest model variable importance ranking. BMI, body mass index; CHD, coronary heart disease; BP, blood pressure; TG, triglyceride; TC, total cholesterol; LDL-C, low-density lipoprotein cholesterol C; HDL-C, high-density lipoprotein cholesterol C; LVEF, left ventricular ejection fraction.

**Figure 2:**
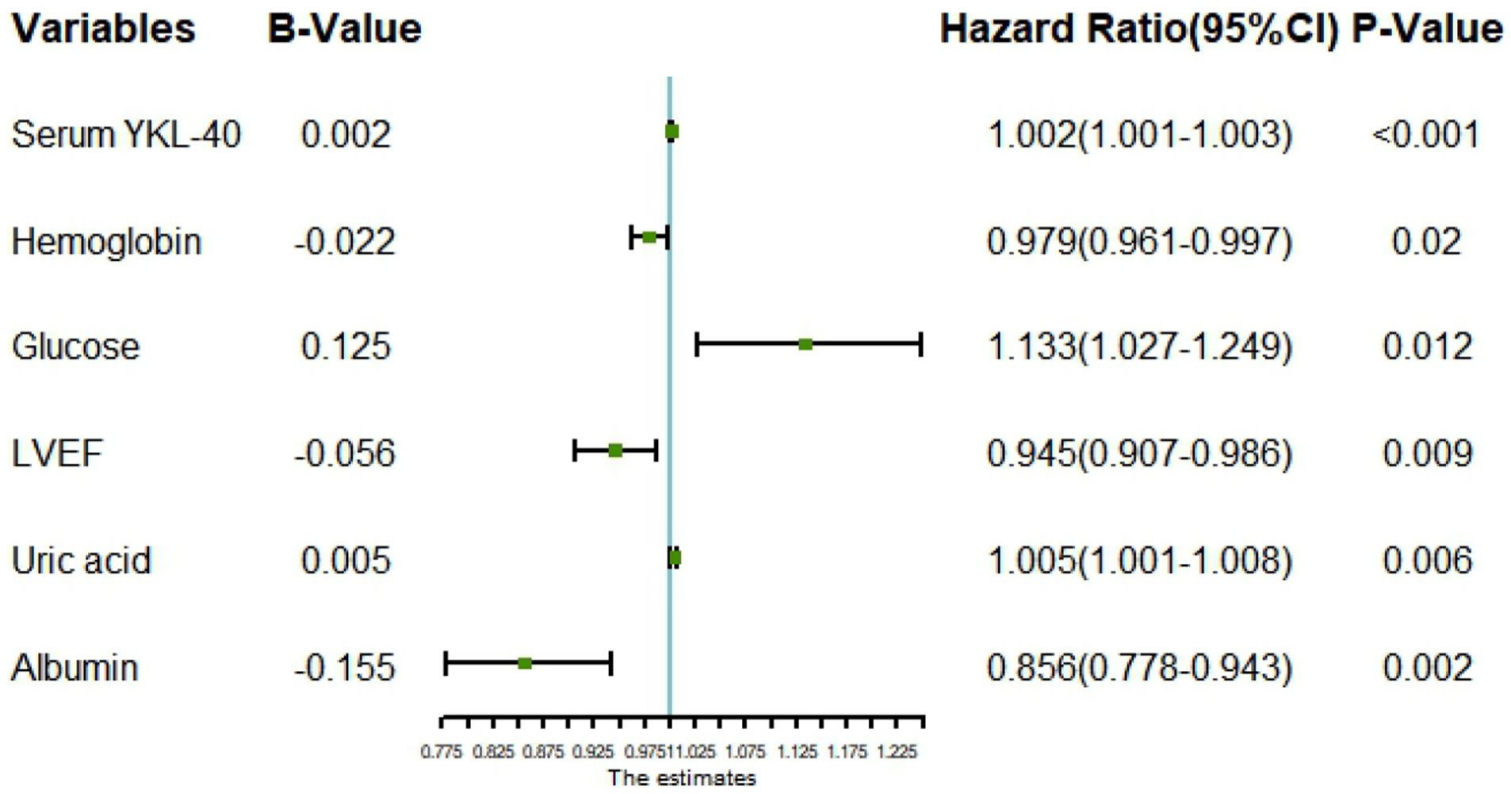
Forest plot for multivariate logistic regression analysis. LVEF, left ventricular ejection fraction.

All variables were included in the RF model to find the optimal RF hyperparameter mtrys of 2, the minimum number of trees of 20, and the total number of trees of 500; the importance ranking of variables was selected using a cross-grid search method (Figure 1); finally the top ten cross-overlapping variables of “MeanDeceAccurscy” and “DecreaseGini” in the importance ranking of RF variables were included in the multivariate logistic regression analysis; the results showed that serum YKL-40, albumin, blood glucose, hemoglobin, LVEF, and uric acid were independent predictors of in-hospital MACE in STEMI patients (Figure 2).

#### 3.3.2. Development of nomogram based on serum YKL-40

**Figure 3:**
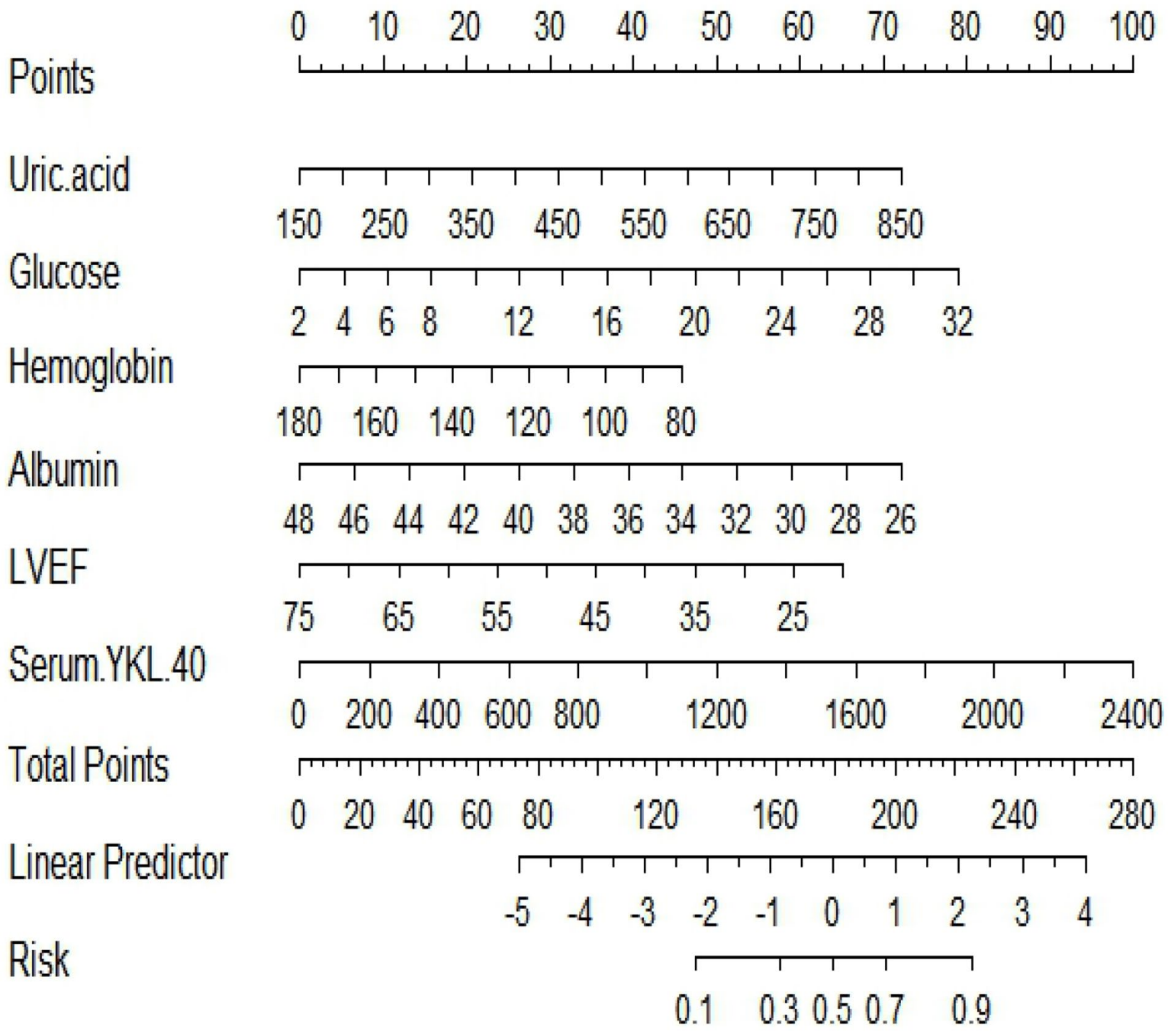
A nomogram based on serum YKL-40 to predict in-hospital major adverse cardiovascular events (MACEs) in patients with ST-segment elevation myocardial infarction (STEMI). LVEF, left ventricular ejection fraction.

Predictive model nomograms for in-hospital MACE in STEMI patients were plotted with multivariate logistic regression analysis using the “RMS” package in R 4.2.2 with whether STEMI patients developed in-hospital MACE (assigned values: no = 0, yes = 1) as the dependent variable, as shown in Figure 3. Each predictor variable corresponds to a specific score on the horizontal axis of the nomogram score, and the score corresponding to each predictor variable is summed to obtain a total score. Through the total score corresponding to the prediction value of the risk of adverse cardiovascular events at the bottom of the nomogram, it can be seen from the figure that the patients with higher total scores are more likely to have in-hospital MACE.

#### 3.3.3. Evaluation of nomogram model based on serum YKL-40

**Figure 4:**
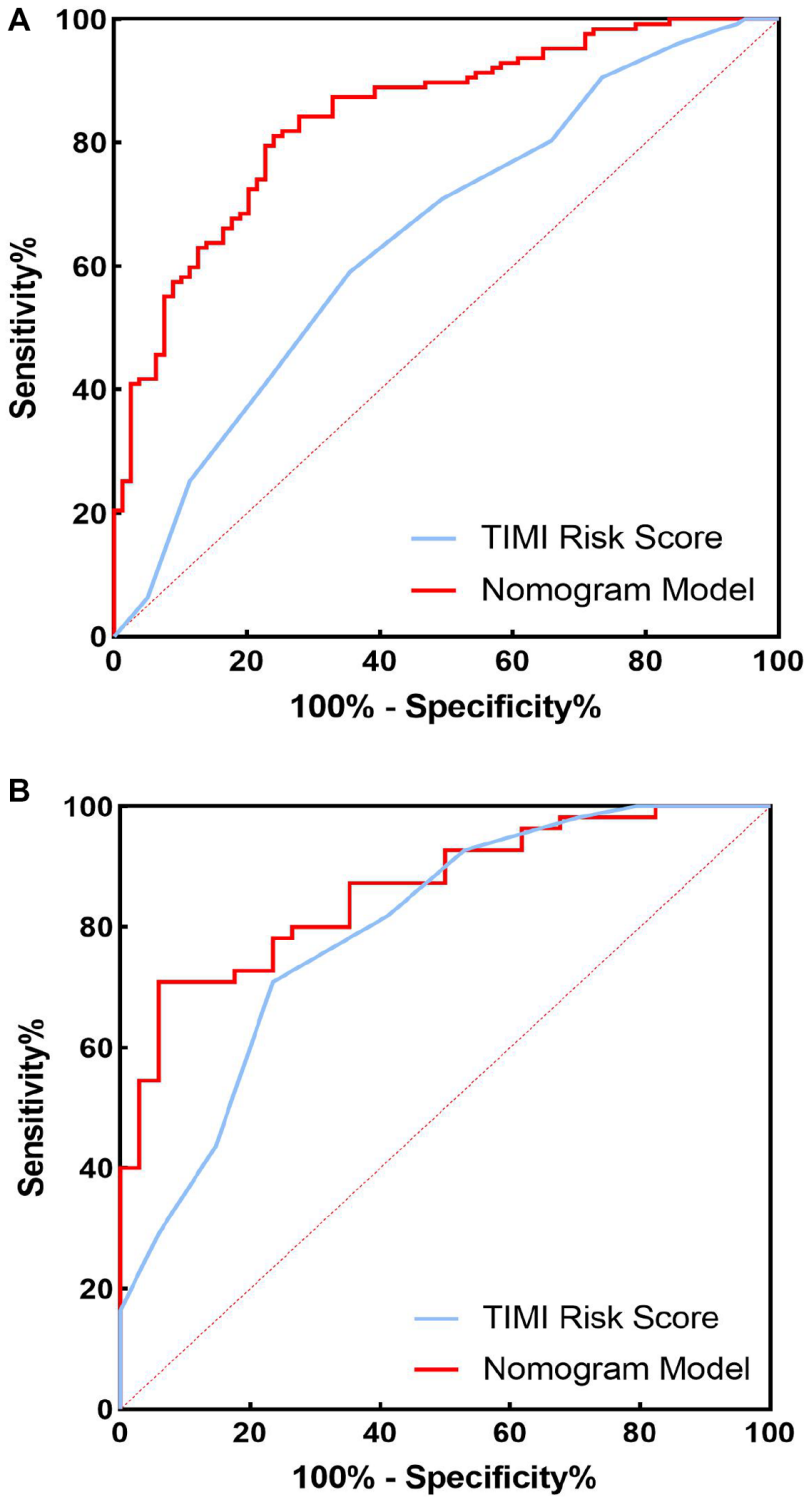
Receiver operating characteristic (ROC) curves of training set and validation set nomogram models and TIMI scores for predicting in-hospital major adverse cardiovascular events (MACEs) in patients with ST-segment elevation myocardial infarction (STEMI). **(A)** Training set; **(B)** validation set.

**Figure 5:**
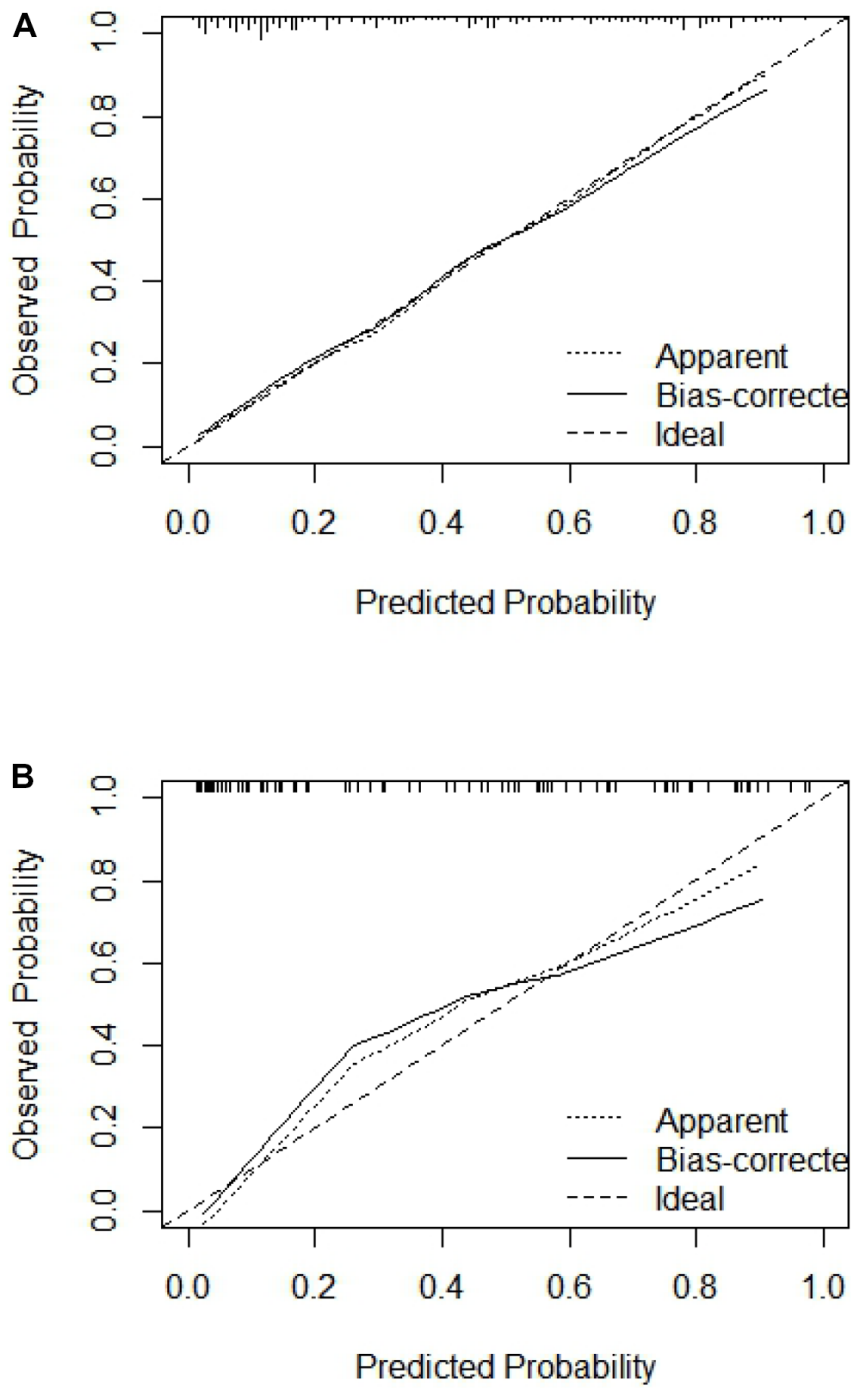
Calibration curve of training set and validation set. **(A)** Training set; **(B)** validation set.

**Figure 6:**
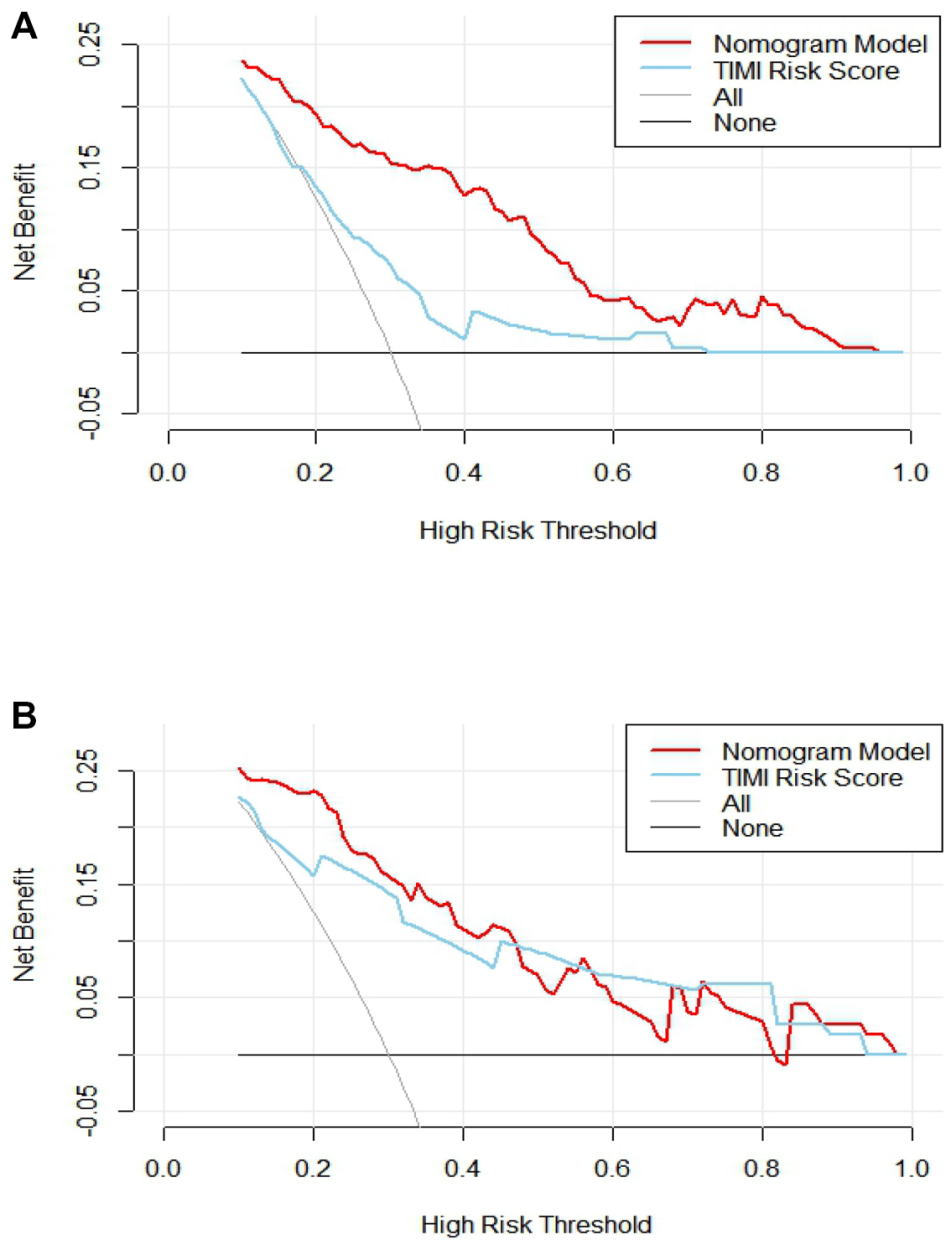
Analysis of clinical decision curve between training set and validation set. **(A)** Training set; **(B)** validation set.

The C-index of the model in the training set was 0.843 (95% CI, 0.79–0.897), which had good predictive power; the AUC of the nomogram model in predicting in-hospital MACE in STEMI patients was 0.843, indicating that the model had good discrimination, as shown in Figure 4A; the calibration curve showed that the model was not fitted, as shown in Figure 5A; and the clinical decision curve analysis (DCA) showed that the clinical net benefit of the nomogram model was superior to the TIMI risk score between 0.1–99% threshold probability, as shown in Figure 6A.

#### 3.3.4. Validation of nomogram model based on serum YKL-40

In the training validation set, the model C-index was 0.863 (95% CI: 0.789–0.936), with good predictive power; the AUC of the nomogram model in predicting in-hospital MACE in STEMI patients was 0.795, with good discrimination, as shown in Figure 4B; the calibration curve also showed that the model had good calibration, as shown in Figure 5B; the clinical decision curve analysis (DCA) showed that the nomogram model had better clinical net benefit than TIMI risk score between 0.1 and 45% threshold probability, as shown in Figure 6B.

#### 3.3.5. Comparison of nomogram models with TIMI risk scores

In the training and validation sets, we compared nomogram models with TIMI risk scores. The AUC of the nomogram model in the training set to predict in-hospital MACE in STEMI patients was 0.843 (0.843) greater than the TIMI score (0.648), *p* < 0.05, as shown in Figure 4A. The AUC of the validated central nomogram model for predicting in-hospital MACE in STEMI patients (0.843) was greater than that of the TIMI score (0.648), *p* < 0.05, as shown in Figure 4B; the results showed that the nomogram had the strongest discriminant ability.

## 4. Discussion

Cardiovascular disease (CVD) has become the focus of global public health problems and the greatest threat to human health (20). In recent years, the incidence and mortality of CVD have been increasing year by year, and are also the most important cause of death among urban and rural residents in China (21). The most important disease in CVD is acute myocardial infarction (AMI), which has high mortality and morbidity (22). AMI refers to myocardial cell death or necrosis due to acute, severe, and persistent ischemia and hypoxia after coronary artery occlusion. It is the most severe subtype of coronary heart disease (23). According to the NHANES database from 2013 to 2016, the overall prevalence of AMI in adults aged >20 years in the US is 3% (24). In addition, AMI has a poor prognosis with a 5-year mortality rate as high as 51%, placing a heavy burden on health and socio-economic burden (25). AMI has a wide spectrum of clinical manifestations, including STEMI and NSTEMI, with high morbidity and mortality. STEMI is a typical symptom, mainly caused by acute intracoronary thrombosis and stagnation of coronary flow, and is the most severe type of AMI with a poor prognosis (26), rapid progression, and high in-hospital mortality (27, 28). Therefore, applying the correct treatment is another key factor in improving the prognosis of STEMI and requires early treatment. Effective early reperfusion therapy to save dying myocardium is a critical component of STEMI treatment and is effective in improving survival and prognosis (29). Current treatments for STEMI patients include thrombolytic therapy, PCI, and coronary artery bypass grafting (CABG). According to European and American clinical guidelines, primary PCI has become the preferred reperfusion therapy strategy for STEMI patients (30). However, even if these patients receive timely PCI and/or appropriate antiplatelet therapy, the prognosis remains unsatisfactory, and a large number of STEMI patients still have a high incidence of MACE during hospitalization after PCI, including cardiac death, myocardial reinfarction, malignant arrhythmia, and acute heart failure (31). Therefore, a new biomarker that may predict the risk of in-hospital MACE after PC in STEMI patients is urgently needed, and further improving the management of STEMI patients during hospitalization is essential to achieve better clinical outcomes in STEMI patients.

Serum YKL-40 is a glycoprotein belonging to the chitinase-similar protein family, also known as human glycochondroprotein 39 (HC-gp39) and chitinase 3-like protein 1 (4). YKL-40 can be secreted by a variety of cells, and current studies have confirmed that it is mainly secreted by macrophages in the late stage of differentiation and activated macrophages (7). The acute phase protein YKL-40 is a novel potential biomarker of inflammation in patients with coronary heart disease, and macrophages in atherosclerotic plaques express YKL-40, with the highest expression seen in macrophages with early atherosclerotic lesions. Studies have shown that serum YKL-40 is elevated in patients with acute myocardial infarction and chronic coronary heart disease (11, 32). The results of previous studies showed (33) serum YKL-40 had prognostic significance in AMI patients, plasma YKL-40 was significantly increased in AMI patients and remained higher than that in healthy subjects after 1 month, and was associated with elevated serum BNP, diastolic dysfunction, and long-term increased overall mortality. Yang et al. (14) further investigated patients with acute STEMI who underwent primary percutaneous coronary intervention (PCI) and were followed for 24 months. Found that the incidence of MACE was significantly higher in the high YKL-40 group than in the low YKL-40 group during follow-up. Therefore, high serum YKL-40 levels are an independent predictor of MACE, and serum YKL-40 can be used as a biomarker to predict the long-term prognosis of STEMI patients after PCI.

To better evaluate the prognosis of patients, the random forest model was used to screen the characteristic variables in this study, and finally, the top ten cross-overlapping variables of “MeanDecreaseAccurscy” and “MeanDecreaseGini” in the importance ranking of random forest variables were included in multivariate logistic regression analysis; the results showed that serum YKL-40 (OR: 1.002, *p* < 0.001), albumin (OR: 0.856, *p* = 0.002), blood glucose (OR: 1.133, P0.012), hemoglobin (OR: 0.979, *p* = 0.02), LVEF (OR: 0.945, *p* = 0.009), and uric acid (OR: 1.005, *p* = 0.006) were independent predictors of in-hospital MACE in STEMI patients. On this basis, we constructed a nomogram prediction model for in-hospital MACE in STEMI patients.

The results of this study showed that serum YKL-40 levels were significantly increased in patients in the MACE group compared with those in the non-MACE group. By RF and multivariate logistic regression analysis, our study identified serum YKL-40 as an independent risk factor for MACE during hospitalization after PCI in STEMI patients, a result consistent with previous studies (16). Consistent with the existing cardiovascular disease literature (10, 14–16), we found that STEMI patients with higher serum YKL-40 levels tended to be older, male, diabetic, family history of coronary heart disease, anterior myocardial infarction, had a higher proportion of multivessel disease, and greater Gensini score. All these features suggest that elevated serum YKL-40 levels may influence the prognosis of STEMI.

Uric acid is the end product of endogenous and dietary purine metabolism and is produced by purine metabolism by xanthine oxidase. Studies have shown the predictive value of uric acid in the hospitalization of patients with the acute coronary syndrome (34). However, not all epidemiological studies support this hypothesis (35). In some studies, uric acid levels were no longer associated with coronary heart disease after additional adjustments for cardiovascular risk factors (36). Some authors consider hyperuricemia as a risk factor rather than an independent risk factor (37). However, other studies have shown that elevated uric acid levels independently predict adverse clinical outcomes after acute myocardial infarction, including increased coronary lesion severity, and mortality (38). High uric acid levels at admission are an independent prognostic factor for adverse outcomes during hospitalization for PCI in patients with acute myocardial infarction (39). Similarly, our study suggests that serum uric acid is associated with the development of in-hospital MACE after PCI in STEMI patients and is an independent risk factor.

Our study found that hemoglobin was an independent predictor of in-hospital MACE after PCI in STEMI patients by multivariate logistic regression analysis. Anemia is more common in patients hospitalized for cardiovascular disease than in the general population, occurring in 11 to 38% of patients with acute myocardial infarction (40). Hemoglobin (Hb) levels on admission are considered a major determinant of mortality and adverse ischemic and hemorrhagic events in patients with cardiovascular disease, particularly ACS (41–43). Anemia at admission has been reported to affect 25% of patients with acute coronary syndromes and has a negative impact on prognosis (44). In a study by Tsujita et al. involving 3,153 STEMI patients (45), anemia was an independent predictor of reinfarction, hemorrhage, and 1-year mortality.

Epidemiological studies have shown that hyperglycemia is an independent risk factor for poor prognosis in patients with acute myocardial infarction regardless of a history of diabetes. Admission stress hyperglycemia has been shown to be associated with increased in-hospital mortality and late follow-up MACE in non-diabetic patients with myocardial infarction (46). Hyperglycemia at admission is strongly associated with mortality, infarct size, impaired left ventricular function, and poor clinical outcome in acute myocardial infarction, and blood glucose at admission is linearly associated with AMI mortality (47). Elevated fasting blood glucose is common in patients with acute myocardial infarction and is significantly associated with increased short-term mortality. In a prospective study, fasting plasma glucose between 6.1 and 6.9 mmol/L was significantly associated with an increased risk of acute myocardial infarction (48). Logistic multivariate regression analysis in this study showed that fasting glucose was an independent risk factor for MACE after PCI in STEMI patients, consistent with previous studies.

Patients with acute myocardial infarction complicated by heart failure (HF) or left ventricular dysfunction have a poor prognosis and are at high risk of rehospitalization and death (49). The use of LVEF measured by echocardiography after acute myocardial infarction to evaluate left ventricular function is an important indicator for predicting clinical prognosis and can well distinguish between low and high risk of cardiac events after acute myocardial infarction. In a study of 417 patients with AMI, LVEF <40% was an independent predictor of the combined end point of death, congestive heart failure, and recurrent AMI 30 years after AMI (50). Another study (51), involving 28,771 patients with HF, left ventricular dysfunction, or both after acute myocardial infarction, showed that the risk of death increased with decreasing LVEF for all types of death. Logistic multivariate regression analysis in this study showed that LVEF was an independent risk factor for MACE after PCI in STEMI patients, consistent with previous studies.

A prospective cohort study (*n* = 734) divided patients with stable coronary artery disease (CAD) into low serum albumin (baseline albumin concentration <3.5 g/dL, *n* = 98) and normal albumin (baseline albumin concentration ≥3.5 g/dL, *n* = 636) groups (52). Low serum albumin concentrations (<3.5 g/dL) have a poor prognosis, with an increased risk of all-cause mortality and cardiovascular events. Another study investigated the effect of low serum albumin levels (*n* = 35) in 82 patients with the acute coronary syndrome (ACS) (in-hospital mortality 10%, 8 patients) and showed that adverse in-hospital outcomes (death, acute heart failure, cardiogenic shock, and re-infarction 43%) were more common in patients presenting with hypoalbuminemia (53). Additional cohort studies have shown that low serum albumin levels on admission are an independent predictor of long-term all-cause, cardiovascular, and cardiac mortality (54). Similarly, a retrospective study of 1,424 patients with acute myocardial infarction showed a higher incidence of in-hospital MACE in patients with low serum albumin levels (55). Our study found that serum albumin was an independent predictor of in-hospital MACE after PCI in STEMI patients by multivariate logistic regression analysis.

**Figure 7:**
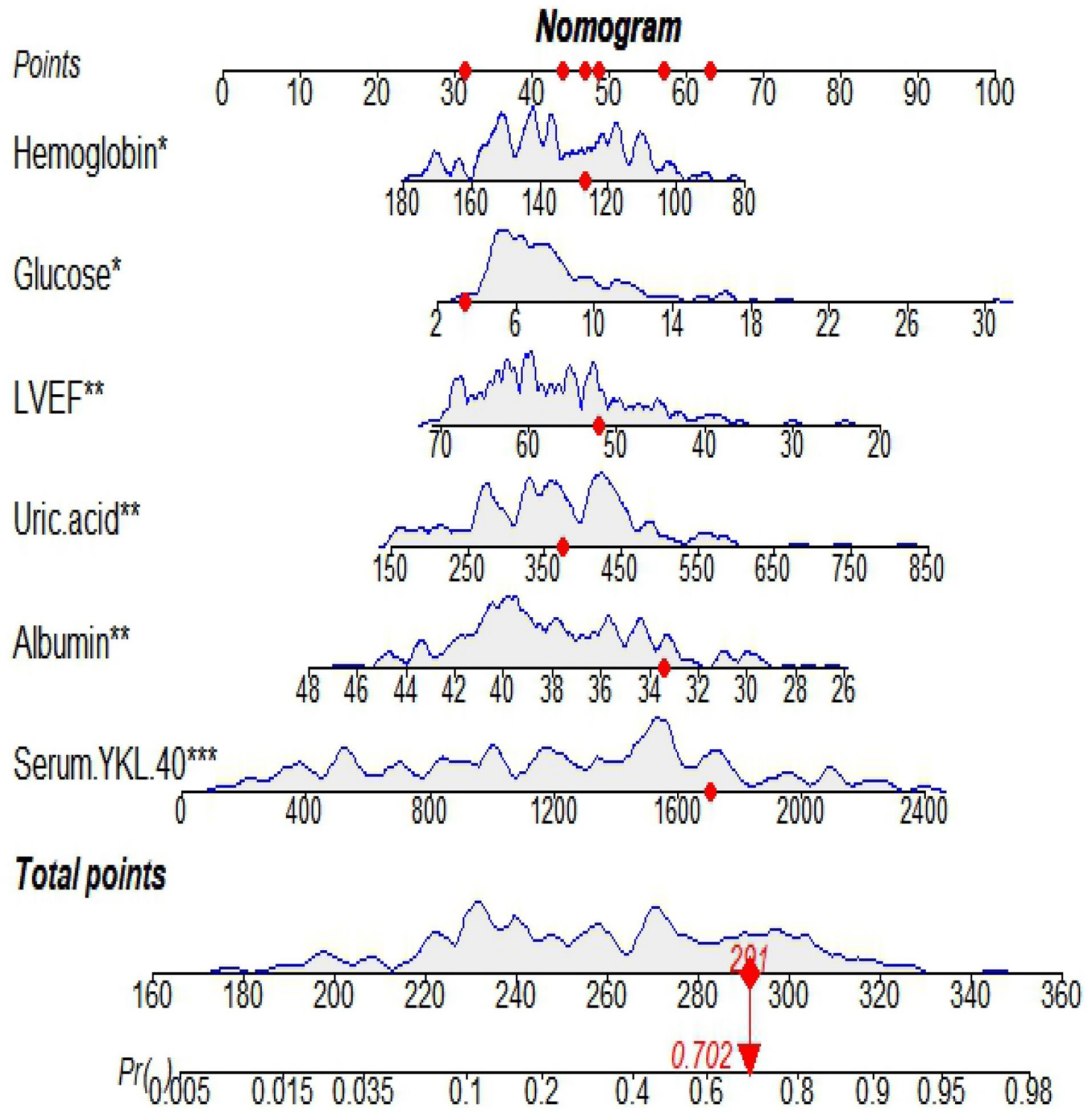
An example of in-hospital MACE nomogram in patients with acute ST-segment elevation myocardial infarction (STEMI).

A nomogram is a visual graph composed of line segments of different lengths that are used to predict the probability of a clinical event, is based on a multivariate regression model, and is drawn after integrating multiple clinical indicators. By RF and multivariate logistic regression analysis, we developed a nomogram containing serum YKL-40, hemoglobin, fasting blood glucose, LVEF, uric acid, and serum albumin variables to predict the risk of in-hospital MACE in STEMI patients. For example, serum YKL-40 was 1706.89 ng/dL, hemoglobin 126.7 g/L, fasting blood glucose 3.39 mmol/L, LVEF 52, uric acid 373.2 umol/L, and serum albumin 33.4 g/L in STEMI patients after admission. The nomogram model calculated a total score of 291 and a 70.2% probability of having MACE during hospitalization (Figure 7).

To further evaluate the predictive value of nomograms for the development of MACE during hospitalization in STEMI patients. We used a risk assessment scoring system to assess whether STEMI patients developed MACE during hospitalization, mainly including the TIMI risk score (56), GRACE score (57), and PAMI risk score (58), of which TIMI risk score is widely used in clinical practice (59, 60). The TIMI score was based on data from the TIMI-Π study, which included patients with STEMI who underwent thrombolytic therapy and mainly analyzed clinical characteristics affecting the risk of death. However, other MACEs also predict adverse outcomes and are more common in clinical practice. Therefore, the AUC of the nomogram model in the training set for predicting in-hospital MACE in STEMI patients was 0.843 (0.843) greater than the TIMI risk score (0.648). Validation of the centralized nomogram model predicted a greater AUC (0.843) than the TIMI risk score (0.648) for in-hospital MACE in STEMI patients; the results indicated that the nomogram had the strongest discriminant power. In parallel, we performed DCA to evaluate the performance of the model. It showed that the net clinical benefit of the nomogram model was better than TIMI risk in both training and validation sets.

This study has some shortcomings. First, this study was a single-center small sample size study without external validation of the model; at the same time, the possibility of selection bias cannot be ruled out. Second, because only studies of MACE during hospitalization after PCI in STEMI patients have been performed, long-term follow-up is needed to assess the long-term prognostic value of serum YKL-40 in STEMI patients in the future.

## 5. Conclusion

In conclusion, this study suggests that a nomogram model based on serum YKL-40 for predicting the risk of in-hospital MACE in STEMI patients has good discrimination, calibration, and clinical validity and can be used as an effective tool for early clinical prediction of the risk of in-hospital MACE after PCI in STEMI patients.

## Data availability statement

The original contributions presented in the study are included in the article/supplementary material, further inquiries can be directed to the corresponding author/s.

## Ethics statement

Written informed consent was obtained from the individual(s) for the publication of any potentially identifiable images or data included in this article. The study was approved by the Ethics Committee of the Second People ‘s Hospital of Hefei (Approval No: 2020-ke-058). Informed consent was obtained from all patients.

## Author contributions

CF and ZC wrote the main manuscript text. JL, WW, and YW prepared Figures 1–7 and Table 1. JZ put forward constructive comments on the overall conception of the article. We also thank for assistance with data collection. All authors contributed to the article and approved the submitted version.

## Funding

This research is funded by the Natural Science Project of Bengbu Medical College in 2020 (2020byzd322).

## Conflict of interest

The authors declare that the research was conducted in the absence of any commercial or financial relationships that could be construed as a potential conflict of interest.

## Publisher’s note

All claims expressed in this article are solely those of the authors and do not necessarily represent those of their affiliated organizations, or those of the publisher, the editors and the reviewers. Any product that may be evaluated in this article, or claim that may be made by its manufacturer, is not guaranteed or endorsed by the publisher.
